# Maternal Exposure to Sulfur Dioxide and Risk of Omphalocele in Liaoning Province, China: A Population-Based Case-Control Study

**DOI:** 10.3389/fpubh.2022.821905

**Published:** 2022-05-12

**Authors:** Li-Li Li, Yan-Hong Huang, Jing Li, Shu Liu, Yan-Ling Chen, Cheng-Zhi Jiang, Zong-Jiao Chen, Yan-Yan Zhuang

**Affiliations:** ^1^Department of Children's Health Prevention, Shenyang Maternity and Child Health Hospital, Shenyang, China; ^2^Department of Ophthalmology, Shenyang Women's and Children's Hospital, Shenyang, China; ^3^Department of Science and Education, Shenyang Maternity and Child Health Hospital, Shenyang, China; ^4^Department of Atmospheric Environment Monitoring, Liaoning Eco-Environmental Monitoring Center, Shenyang, China; ^5^Office of Institution, Liaoning Women and Children's Health Hospital, Shenyang, China; ^6^School of Environmental and Chemical Engineering, Shenyang Ligong University, Shenyang, China; ^7^Department of Obstetrics and Gynecology, Shengjing Hospital of China Medical University, Shenyang, China

**Keywords:** air pollution, case-control study, omphalocele, risk, sulfur dioxide

## Abstract

Evidence of the association between maternal sulfur dioxide (SO_2_) exposure and the risk of omphalocele is limited and equivocal. We aimed to assess the aforementioned topic during the first trimester of pregnancy. A population-based case-control study was carried out in infants consisting of 292 cases of omphalocele and 7,950 healthy infant controls. Exposure to SO_2_, particulate matter with aerodynamic diameters ≤ 10 μm, and nitrogen dioxide was assessed by averaging the concentration from all stations in the mother's residential city. SO_2_ exposure was categorized into three groups, with the lowest tertile defined as the reference category. Odds ratios (ORs) and 95% confidence intervals (CIs) were estimated using multivariable logistic regression models. Higher SO_2_ exposure during the first trimester was significantly associated with omphalocele risk [per standard deviation (42 ug/m^3^) increment: OR = 1.39, 95% CI = 1.22–1.65]. When focusing on shorter exposure windows, similar positive associations were observed for SO_2_ exposure in the first and third months of pregnancy. In addition, compared with the lowest tertile, high SO_2_ exposure in the second month of pregnancy increased the risk of omphalocele (OR = 2.80, 95% CI = 1.61–4.97). Maternal exposure to SO_2_ during the first trimester may increase the risk of omphalocele in offspring.

## Introduction

Omphalocele is one of the most frequently observed congenital abdominal wall defects, with an incidence rate of ~1–3.8 per 10,000 live births (1–4). In China, the incidence of omphalocele in 2019 was 0.82 per 10,000 births, with no significant changes during recent years (5). In omphalocele, organs protrude through a midline defect in the abdominal wall along with the umbilical cord (6). This can affect not only the small intestine and liver, but also other organs such as the bladder, spleen, and ovaries (7). The survival rate of isolated omphalocele can reach 90%, but is significantly reduced with other malformations (8). Due to lack of a clear consensus to explain the precise embryological mechanisms leading to the occurrence of omphalocele (7), prevention is best achieved by exploring environmental teratogens and providing recommendations to pregnant women at the optimal time window.

Growing epidemiological evidence has shown that maternal exposure to ambient air pollution may have adverse effects on a developing fetus or newborn (9, 10). Several studies from the most recent 5 years have found significant associations between maternal air pollution exposure and increased risk of congenital cardiac defects, neural tube defects, and limb malformations (9–11). Furthermore, findings from our colleagues also suggested that maternal air pollution exposure is positively associated with neural tube defects, oral clefts, polydactyly, and syndactyly (12–17). However, only a few studies focused on the association between ambient air pollution and the risk of congenital abdominal wall defects (18, 19). For example, a population-based case-control study in Barcelona reported that exposure to ambient particulate matter with aerodynamic diameters ≤ 10 μm (PM_10_), PM_coarse_, and PM_2.5_ increased the risk of abdominal wall defects, but exposure to nitrogen dioxide (NO_2_) and nitrous oxide had no effect (18). However, previous studies have found that maternal exposure to sulfur dioxide (SO_2_) is not associated with the risk of multiple birth defects (19, 20). A previous geographical study in England failed to observe a significant relationship between maternal SO_2_ exposure and omphalocele risk (19). In addition, no significant association of maternal SO_2_ exposure during the first trimester was observed among 75 omphalocele cases in Xi'an from 2010 to 2015 (10).

In light of the inadequate evidence on the risk of omphalocele in the different stages of pregnancy as well as the lack of studies in China, and based on data from the Liaoning Province, we conducted a population-based case-control study to explore whether exposure to ambient SO_2_ during the first 3 months of pregnancy was associated with omphalocele risk.

## Methods

### Study Populations and Data Sources

Data on live births with omphalocele were obtained between 1 January 2010 and 31 December 2015 from the Maternal and Child Health Certificate Registry of Liaoning Province, which was managed by Liaoning Women and Children's Health Hospital, Shenyang, China. Several previous studies have described this registry in detail (21). In short, the registry is a hospital-based active monitoring system for monitoring live births. The hospital is a large obstetrics and gynecology hospital and a comprehensive nursing institution. Liaoning Maternal and Child Health Care Guidance Center has provided comprehensive health care services for pregnant women since 1986. A total of 14 cities (Shenyang, Dalian, Anshan, Fushun, Benxi, Dandong, Jinzhou, Yingkou, Fuxin, Liaoyang, Panjin, Tieling, Chaoyang, and Huludao) in Liaoning Province have been registered. During the study period, there were ~6,000 cases of birth defects across all obstetric units each year (22). Liaoning is one of 31 provinces that provides data to the national birth defects monitoring database, which is maintained by the Chinese Birth Defects Monitoring Network. Fourteen maternal and child health care institutions in Liaoning Province provide birth defect data to the Liaoning Women and Children's Health Hospital each month (23). We have previously described the geographical divisions of Liaoning Province and the source of the control group (14). The control group was constructed to represent the general population of births in Liaoning Province, from where the case groups were recruited. Therefore, 1.5% of live births without birth defects were randomly selected from five cities (Shenyang, Dalian, Fuxin, Chaoyang, and Huludao) as a control of random birth year sampling unrelated to cases from 2010 to 2015. If the subject did not have a permanent address or if key covariates were missing, they were excluded from the current analysis. The study protocol has been approved by the Institutional Review Board of Liaoning Women and Children's Health Hospital and carried out in accordance with local and national regulations.

### Data Collection and Quality Control

Several previous studies had described the data collection procedure (21). In short, provincial and municipal monitoring networks and clinical expert groups have been established to collect data. An experienced obstetrician or pediatrician examined each newborn (or terminated fetus) immediately after birth. Suspected cases of omphalocele that were diagnosed by prenatal ultrasonography were confirmed after termination or postnatal examination. Omphalocele (ICD-10-CM code, Q79.2) were registered and coded according to the International Classification of Diseases 10th edition. After identifying and confirming omphalocele cases in the hospital, an experienced obstetric or pediatric expert interviewed the mother of the infant and completed the Birth Defects Registration Form, which was easily used to collect demographic characteristics, clinical features, and obstetric factors. Subsequently, forms were submitted to the local maternal and child healthcare institution, and then to the Liaoning Women and Children's Health Hospital. The data were reviewed and analyzed by a team of national grade experts in medical genetics and pediatrics (24). We have previously reported quality control data (24); in short, experts at all levels identified the disease diagnosis, data collection, data checking, as well as medical records according to the procedure manual to ensure high quality of data. Additionally, an independent retrospective investigation was conducted by experts to identify inadequacy and inaccuracies in the data (24).

### Air Pollutant Exposure Assessment

**Figure 1:**
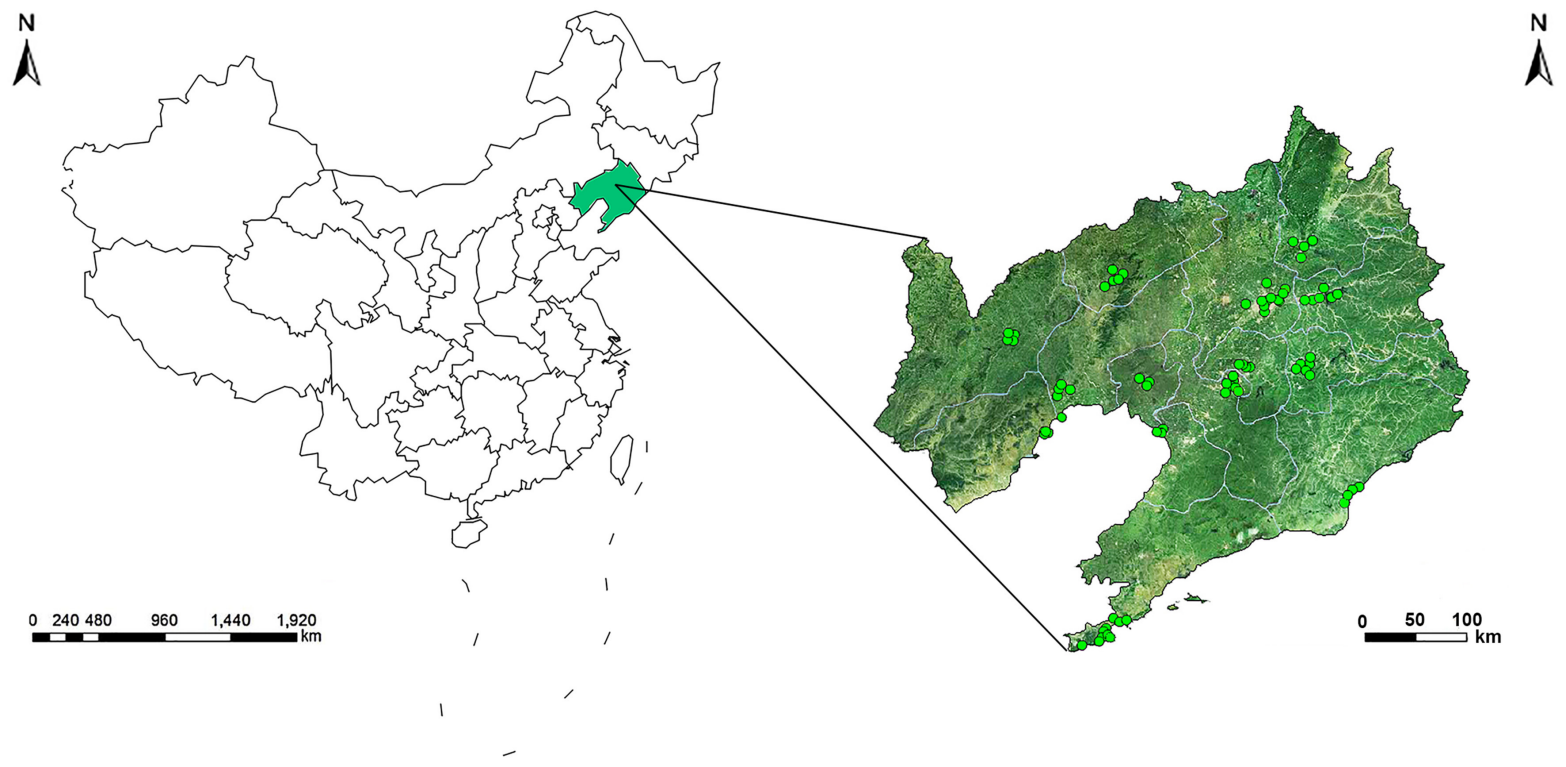
Geographic locations of air monitoring stations in 14 cities in Liaoning Province, China.

The data from 77 air quality monitoring stations in 14 cities of Liaoning Province (two of which served as controls) were used to assess the ambient air pollution exposure during the period of pregnancy (Figure 1). The 77 air pollutant monitoring stations across Liaoning Province were located primarily in urban areas, covering residential areas to represent the ambient air pollution levels of the whole area. We used the above mentioned monitoring stations to measure the daily SO_2_, NO_2_, and PM_10_ levels from 2010 to 2015. The hourly concentration of the monitoring station was tested and reported in strict accordance with the ambient air quality standards of the Chinese government (25). To assess maternal exposure level, we used the average concentrations of SO_2_, NO_2_, and PM_10_ at all sites in the city where the mother lived, and then used these values to calculate an average monthly exposure (Supplementary Figure S1). Environmental SO_2_, NO_2_, and PM_10_ concentrations were available from the Environment Protection Bureau of each city. The monthly averages were linked to birth records according to the city where the mother lived, as indicated on the Birth Defects Registration Form. Most congenital malformations occur in the first trimester of pregnancy, which is a very important stage of development for the baby's organogenesis (17). And we choose the first trimester of pregnancy and their individual month. In order to investigate the exposure window during the period of pregnancy, average SO_2_ air pollution concentrations for every participant were calculated at a time point 3 months after conception. As with previous studies (14, 17), we assumed that the pregnancy date occurred on the first day of the last menstruation, and we calculated the pregnancy date according to the birth date and gestational age information on the form. If the date of pregnancy occurred in the first half of the month, then the month was regarded as the first month after conception; otherwise, it was regarded as the first month before conception.

### Covariates

We identified potential confounders based on a previous causal knowledge of the existing literature (26, 27), including: season of conception [four categories: spring (March–May), summer (June–August), autumn (September–November), and winter (December–February)]; maternal age (three categories: <30 and ≥30 years), parity (≤1 and ≥2), gravidity (≤1 and ≥2); maternal education level (four categories: elementary school or less, middle school, high school, and college or above); and maternal NO_2_ and PM_10_ exposure.

### Statistical Analysis

The characteristics of cases and controls were compared using the chi-square test. The monthly distributions of SO_2_ concentrations in Liaoning Province during the study period were described by mean ± standard deviation and median (25th−75th percentile) to provide an overall view of the characteristics of ambient air pollution in the study area. Omphalocele was treated as a dichotomous dependent variable in the analysis. The exposure concentration of SO_2_ acted as the principal independent variable that was calculated as the categorical variable based on tertiles of distribution in the control groups. We used the lowest tertile as the reference category. Odds ratios (ORs) and 95% confidence intervals (CIs) were calculated through crude and multivariable logistic regression analysis. The models were: without any adjustment (crude); adjusted for maternal age, season of conception, gravidity, parity, and maternal education (model 1); additionally adjusted for NO_2_ on the basis of model 1 (model 2); additionally adjusted for PM_10_ on the basis of model 1 (model 3); and additionally adjusted for NO_2_ and PM_10_ on the basis of model 1 (model 4)_._ In addition, we completed subgroup analysis to assess potential effect modification by maternal age (<30 and ≥30 years). Potential interactions between the concentration of SO_2_ and maternal age were assessed by adding cross-product terms to the multivariable logistic models. All analyses were performed using SAS version 9.4 (SAS Institute Inc., Cary, NC, USA). Statistical significance was set at *P* < 0.05, based on the two-sided test.

## Results

The basic characteristics of cases and controls in Liaoning Province are shown in Table 1. Cases of omphalocele (*n* = 292) and controls (*n* = 7,950) were included in our analysis. Compared with controls, omphalocele cases had younger gestational age, lower birth weight, and more births occurring during fall and winter (*P* < 0.001). There were no significant differences between the two groups with respect to infant sex or maternal age. Table 2 shows the distribution characteristics of ambient SO_2_ concentration (μg/m^3^) in the 14 cities in Liaoning Province from 2010 to 2015. The SO_2_ concentration in Panjin was the lowest during the study period; Shenyang, the capital of Liaoning Province, had the highest SO_2_ concentration and exceeded the recommended annual SO_2_ concentration limit (60 μg/m^3^).

**Table 1:** Characteristics of controls and cases in Liaoning Province, China, 2010–2015 [no. (%)].

**Characteristic**	**Cases**	**Controls**	***P*-value**
Total	292 (100)	7,950 (100)	
**Season of conception**			<0.001
Spring	71 (24.3)	2,106 (26.5)	
Summer	77 (26.4)	2,829 (35.6)	
Autumn	90 (30.8)	1,705 (21.4)	
Winter	54 (18.5)	1,310 (16.5)	
**Gender of infant**			0.55
Female	139 (47.6)	3,927 (49.4)	
Male	153 (52.4)	4,023 (50.6)	
**Gestational age, weeks**			<0.001
<37	207 (70.9)	257 (3.2)	
≥37	85 (29.1)	7,693 (96.8)	
**Birth weight, grams**			<0.001
<2,500	194 (66.4)	174 (2.2)	
2,500–<4,000	90 (30.8)	6,840 (86.0)	
≥4,000	8 (2.7)	936 (11.8)	
**Maternal age, years**			0.84
<30	171 (58.6)	4,704 (59.2)	
≥30	121 (41.4)	3,246 (40.8)	
**Gravidity**			<0.001
≤1	147 (50.3)	5,026 (63.2)	
≥2	145 (49.7)	2,924 (36.8)	
**Parity**			<0.001
≤1	248 (84.9)	7,695 (96.8)	
≥2	44 (15.1)	255 (3.2)	
**Maternal education**			<0.001
Elementary school or less	13 (4.5)	265 (3.3)	
Middle school	129 (44.2)	2,912 (36.6)	
High school	77 (26.4)	1,723 (21.7)	
College or above	73 (25.0)	3,050 (38.4)	

**Table 2:** Ambient SO_2_ levels (μg/m^3^) and the number of air monitoring stations in 14 cities in Liaoning Province, China, between 2010 and 2015.

**Cities**	**Mean ±SD**	**Median (25tile-75tile)**	**Range**	**Number of air monitoring stations**
Shenyang	69 ± 59	41 (27–102)	246	10
Dalian	34 ± 28	22 (11–59)	108	10
Anshan	51 ± 43	28 (17–86)	154	6
Fushun	38 ± 22	31 (21–51)	83	6
Benxi	51 ± 38	41 (19–78)	133	5
Dandong	36 ± 28	20 (15–61)	92	4
Jinzhou	42 ± 29	31 (22–55)	109	6
Yingkou	31 ± 22	22 (14–47)	78	4
Fuxin	47 ± 22	41 (30–60)	91	5
Liaoyang	47 ± 27	38 (28–61)	123	4
Panjin	25 ± 12	22 (17–29)	55	3
Tieling	31 ± 20	25 (15–42)	84	4
Chaoyang	39 ± 26	29 (18–58)	99	4
Huludao	46 ± 28	35 (24–65)	103	4

*SD, standard deviation; tile, percentile*.

Findings of the main analyses are displayed in Table 3. In model 4, which adjusted for maternal age, season of conception, gravidity, parity, nitrogen dioxide, and PM_10_, we found that higher SO_2_ exposure increased the risk of omphalocele [per standard deviation (42 ug/m^3^) increment: OR = 1.39, 95% CI = 1.22–1.65]. Further evaluation by single month showed similar results for the first and third months of pregnancy. In addition, we found that the highest tertile of maternal SO_2_ exposure was associated with an increased risk of omphalocele during the second month of pregnancy (OR _T3vs.T1_ = 2.80, 95% CI = 1.61–4.97).

**Table 3:** Associations between maternal exposure to ambient SO_2_ during various exposure windows and the risk of omphalocele in offspring.

**Tertiles of SO_**2**_ level^a^**	**No. of cases**	**No. of controls**	**Unadjusted OR (95% CI)**	**Model 1^b^ (95% CI)**	**Model 2^c^ (95% CI)**	**Model 3^d^ (95% CI)**	**Model 4^e^ (95% CI)**
**The first month of pregnancy**							
<21	80	2,490	1.00 (ref)	1.00 (ref)	1.00 (ref)	1.00 (ref)	1.00 (ref)
21 to <40	96	2,771	1.08 (0.80–1.46)	1.16 (0.84–1.62)	1.82 (0.85–1.65)	1.22 (0.87–1.71)	1.11 (0.79–1.56)
≥40	116	2,689	1.34 (1.01–1.80)	1.07 (0.71–1.62)	1.76 (1.15–2.70)	1.21 (0.77–1.89)	1.56 (0.99–2.45)
Per 1-SD (47 μg/m^3^) increase			1.11 (1.06–1.18)	1.10 (1.05–1.18)	1.15 (1.07–1.29)	1.12 (1.06–1.22)	1.13 (1.06–1.25)
**The second month of pregnancy**							
<21	73	2,551	1.00 (ref)	1.00 (ref)	1.00 (ref)	1.00 (ref)	1.00 (ref)
21 to <45	88	2,746	1.12 (0.82–1.54)	1.30 (0.93–1.83)	1.47 (1.04–2.07)	1.42 (1.00–2.02)	1.42 (1.00–2.02)
≥45	131	2,653	1.73 (1.29–2.32)	1.80 (1.07–3.07)	2.96 (1.72–5.19)	2.11 (1.23–3.69)	2.80 (1.61–4.97)
Per 1-SD (47 μg/m^3^) increase			1.08 (0.97–1.18)	0.93 (0.79–1.09)	1.17 (1.02–1.35)	1.02 (0.83–1.21)	1.13 (0.96–1.32)
**The third month of pregnancy**							
<23	89	2,468	1.00 (ref)	1.00 (ref)	1.00 (ref)	1.00 (ref)	1.00 (ref)
23 to <52	84	2,825	0.83 (0.61–1.12)	0.80 (0.55–1.14)	0.90 (0.63–1.29)	0.87 (0.60–1.25)	0.90 (0.63–1.30)
≥52	119	2,657	1.24 (0.94–1.65)	0.85 (0.53–1.35)	1.38 (0.85–1.23)	1.01 (0.62–1.63)	1.39 (0.85–2.28)
Per 1-SD (47μg/m^3^) increase			1.12 (1.05–1.21)	1.10 (1.03–1.19)	1.19 (1.07–1.42)	1.13 (1.05–1.30)	1.23 (1.08–1.55)
**The first trimester**							
<24	84	2,406	1.00 (ref)	1.00 (ref)	1.00 (ref)	1.00 (ref)	1.00 (ref)
24 to <50	88	2,912	0.87 (0.64–1.17)	0.82 (0.58–1.17)	1.05 (0.73–1.51)	0.93 (0.64–1.33)	1.04 (0.72–1.52)
≥50	120	2,632	1.31 (0.99–1.74)	0.89 (0.53–1.49)	1.70 (1.00–2.91)	1.25 (0.70–2.24)	1.66 (0.91–3.02)
Per 1-SD (42 μg/m^3^) increase			1.21 (1.12–1.30)	1.21 (1.10–1.33)	1.33 (1.20–1.50)	1.34 (1.20–1.54)	1.39 (1.22–1.65)

*CI, confidence interval; OR, odds ratios; SD, standard deviation; SO2, sulfur dioxide; ref, reference*.

a *SO_2_ concentrations (μg/m^3^) are based on the monthly average concentrations, which are then averaged over different exposure windows and analyzed in tertiles (determined from controls)*.

b *Model 1 adjusted for maternal age, season of conception, gravidity, parity and maternal education*.

c *Model 2 adjusted for covariates in model 1 plus nitrogen dioxide exposure levels during the same period*.

d *Model 3 adjusted for covariates in model 1 plus particulate matter with an aerodynamic diameter ≤ 10 μm exposure levels during the same period*.

e *Model 4 adjusted for covariates in model 1 plus nitrogen dioxide and particulate matter with an aerodynamic diameter ≤ 10 μm exposure levels during the same period*.

Supplementary Table 1 shows the adjusted risk estimates between maternal SO_2_ exposure and omphalocele risk, stratified by maternal age. A significant association between the highest tertile of maternal SO_2_ exposure and omphalocele risk was found during the first trimester in the younger age group. Additionally, no interaction was observed between maternal SO_2_ exposure and maternal age.

## Discussion

In this population-based case-control study, we estimated the association between maternal exposure to air pollutants and the risk of omphalocele in offspring using the database of the Maternal and Child Health Certificate Registry of Liaoning Province from 2010 to 2015. We observed a significant positive association between ambient SO_2_ exposure during the first trimester and omphalocele risk.

The mechanism by which maternal SO_2_ exposure during pregnancy causes omphalocele remains unclear, and further mechanistic research and animal experiments are needed. However, several possible mechanisms involving placental inflammation (28), oxidative stress (39), epigenetic changes (29), and microRNA (30) have been proposed in many epidemiological studies to explain the observed effects birth defects following maternal exposure to environmental pollutants. Specifically, SO_2_ may disrupt the structure of DNA and induce epigenetic changes, such as DNA methylation and histone modifications, which can be passed on to offspring (31). In addition, SO_2_ absorbed into the human body can produce toxic effects on embryonic development, and destroy the function and microstructure of germ cells (32).

To our knowledge, limited air pollution studies (10, 19, 33, 34) have focused on omphalocele as the primary or secondary study outcome. For example, in a previous exploratory investigation in Texas, Vinikoor-Imler et al. found that high maternal PM_2.5_ and O_3_ exposures during the first trimester were not associated with an increased risk of omphalocele in offspring (33). As a rare but serious birth defect, only two published studies (10, 19) have examined the association between maternal SO_2_ exposure during pregnancy and the risk of omphalocele in offspring. Contrary to our results, these two studies did not find a positive association between maternal SO_2_ exposure during the first trimester and omphalocele risk. Inconsistencies may be attributed to differences in study design (time-series study, ecological study, or case-control study), statistical analysis methods (generalized additive model, Poisson regression model, or logistic regression model), exposure assessment methods, sample size, and adjustments for confounding factors. For example, Dolk et al. (19) performed a geographical study analyzing a population-based active surveillance database of birth defects across four regions of England from 1991 to 1999 to estimate associations between average annual exposure to SO_2_, NO_2_, and PM_10_ and broad groups of birth defects. A total of 183 cases with omphalocele were included for final poisson regression analysis, and the results reported a significant positive association between maternal PM_10_ exposure during the first trimester and omphalocele risk. However, the same multivariable logistic regression model showed no association between maternal SO_2_ or NO_2_ exposures during the first trimester and the risk of omphalocele. Wang et al. (10) used a time-series design to investigate the associations between various types of birth defects and maternal SO_2_, NO_2_, and PM_10_ exposures during early pregnancy in Xi'an city, China, from 2010 to 2015. However, this study did not observe any significant positive association between the risk of omphalocele (*n* = 75) and SO_2_ exposure during the first 3 months of pregnancy.

Our study has several advantages. First, the sample size included in the final analysis was larger than in the two previous studies, which enabled us to examine the association between SO_2_ exposure during the first trimester and the risk of omphalocele in a more statistically precise manner. Second, our provincial birth registry database recruited not only live births with omphalocele, but also stillbirths and aborted fetuses with omphalocele, which increased the number of cases and reduced possible selection bias.

Our findings should be interpreted carefully and are not without limitation. First, the method of exposure assessment may have masked the true associations. In our study, we assigned to each subject average air pollutant levels for all air quality monitoring stations of maternal residential areas, which may have created exposure misclassification. Due to lack of data on land use and transportation, we were not able to evaluate maternal SO_2_ exposures using the land-use regression model. Future air pollution studies with birth defects as the primary or secondary health outcome should emphasize the application of accurate exposure assessments to reduce measurement errors that may arise from exposure assessments. Second, the possible movement or relocation of a pregnant woman during pregnancy also affects the concentration of exposure assessed from fixed air quality monitoring stations. Investigators typically survey mothers after birth and may overlook differences in residence changes between early pregnancy and delivery. A review (35) of 14 air pollution studies with maternal residential mobility data available for the entire pregnancy reported that 9–32% gravidae change residence between conception and delivery, and pregnant women generally maintain a low mobility rate during the early stages of pregnancy. Notably, the results of two large air pollution studies conducted in China (36, 37) showed that the mobility rate of Chinese women during pregnancy is about 3%. Therefore, errors in exposure assessments due to changes in residence are unlikely to have a significant effect on the associations of maternal air pollutants exposures with birth defects. Third, our data were collected from the birth registrations. Although patients with birth defects were recorded by active surveillance and rigorous quality control, unreported and misclassified omphalocele cases are inevitable, especially in areas with limited medical resources (38). Fourth, although we adjusted some important confounding factors based on our experience and previous studies, the influence of residual confounding factors on our results cannot be completely excluded. Due to the availability of data, we were unable to adjust for some maternal environmental exposure factors, including maternal illness, maternal smoking or alcohol intake, medication exposures, and maternal nutritional intake during early pregnancy. However, it is unlikely that such factors were associated with exposure to air pollutants and were partially controlled by adjusting for family income or maternal education level in the multivariable logistic regression model (15). Fifth, since PM_2.5_ monitoring began in 2013 in Liaoning Province and there were insufficient data available, we cannot assess the effects of PM_2.5_ in our models. Future studies should attention smaller particles.

## Conclusion

In conclusion, our study found that high maternal SO_2_ exposure during the first trimester is associated with an increased risk of omphalocele in offspring. We recommend that women in early pregnancy should avoid or reduce exposure to air pollutants; further mechanistic studies are necessary to confirm the associations identified in this population study.

## Data Availability Statement

The original contributions presented in the study are included in the article/Supplementary Material, further inquiries can be directed to the corresponding author.

## Ethics Statement

The studies involving human participants were reviewed and approved by Liaoning Women and Children's Health Hospital. Written informed consent for participation was not required for this study in accordance with the national legislation and the institutional requirements.

## Author Contributions

Y-YZ and Y-HH: study conceptualization and design. JL and SL: data collection. Y-YZ: data cleaning and discrepancy checks. Y-LC and C-ZJ: analytic strategy. L-LL and Z-JC: analysis and interpretation of data. L-LL, JL, and SL: manuscript preparation. All authors read and approved the final manuscript.

## Funding

This study was supported by the Liaoning Providence Science and Technology Project (2015225025 for Y-HH), the Shenyang science and technology project (F15-139-9-09 for Y-HH).

## Conflict of Interest

The authors declare that the research was conducted in the absence of any commercial or financial relationships that could be construed as a potential conflict of interest.

## Publisher's Note

All claims expressed in this article are solely those of the authors and do not necessarily represent those of their affiliated organizations, or those of the publisher, the editors and the reviewers. Any product that may be evaluated in this article, or claim that may be made by its manufacturer, is not guaranteed or endorsed by the publisher.

## Supplementary Material

Supplementary Figure S1 Monthly mean ambient SO_2_ concentrations (ugm^3^) in 14 cities in Liaoning Province, China.

Supplementary Table 1 The maternal age stratification (<30,≥30 years) subgroups were analyzed for the associations between ambient SO_2_ exposure(μg/m^3^) with controls and cases.

## Abbreviations

CIs: confidence intervals
NO_2_: nitrogen dioxide
ORs: odds ratios
O_3_: ozone
PM_10_: particulate matter with aerodynamic diameter ≤ 10 μm
PM_2._5: particulate matter with aerodynamic diameters ≤ 2.5 μm
SD: standard deviation
SO_2_: sulfur dioxide
Tile: percentile.
