# Supplementary figures and images for: Maternal Exposure to Sulfur Dioxide and Risk of Omphalocele in Liaoning Province, China: A Population-Based Case-Control Study
Source: Front Public Health. 2022 May 12;10:821905. doi: 10.3389/fpubh.2022.821905 (PMC9133471; doi:10.3389/fpubh.2022.821905)

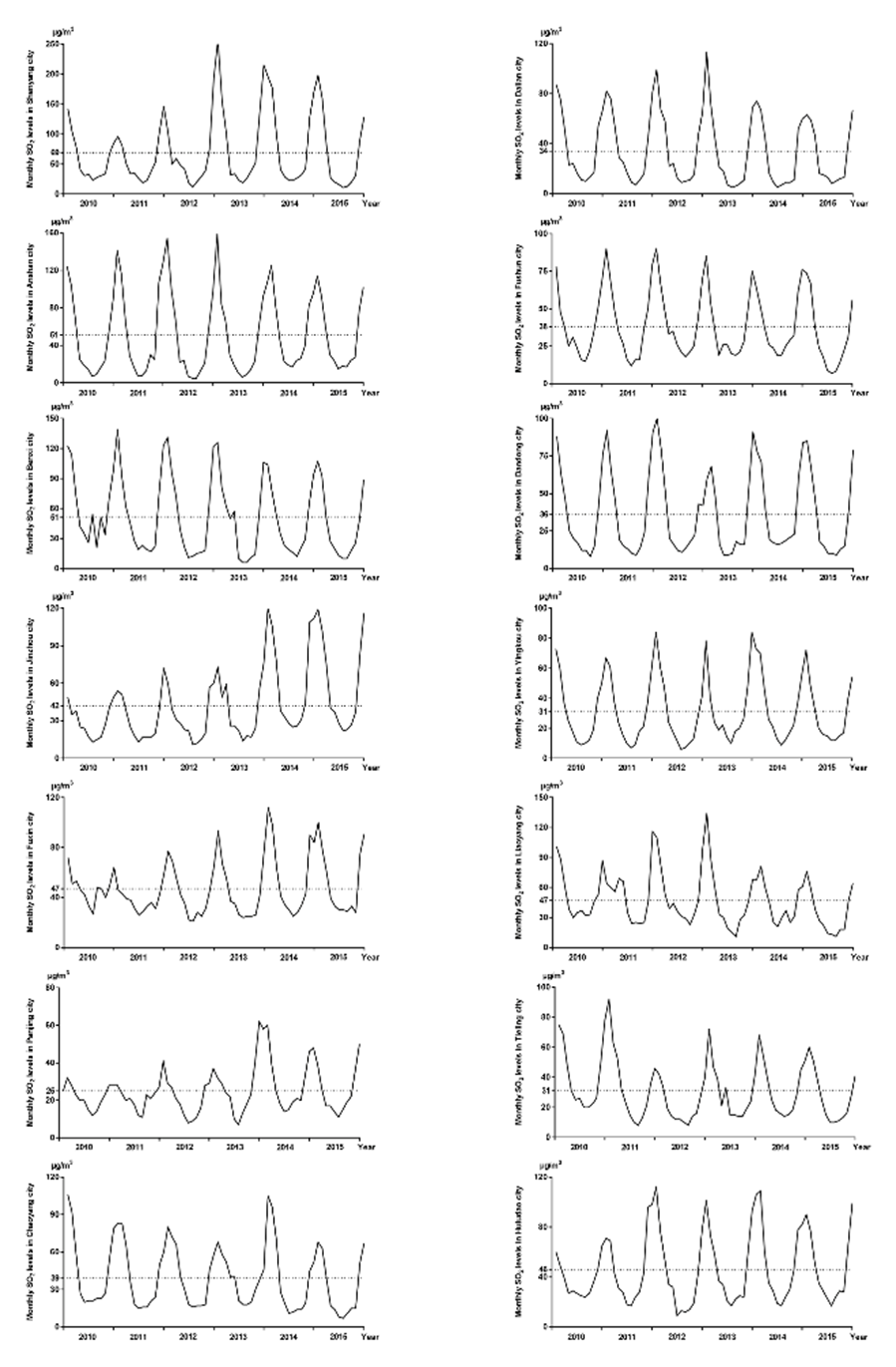

Supplement: Supplementary Figure S1 — Monthly mean ambient SO2 concentrations (ugm3) in 14 cities in Liaoning Province, China. [file Image_1.tif]
