# Supplementary material for: Maternal Exposure to Sulfur Dioxide and Risk of Omphalocele in Liaoning Province, China: A Population-Based Case-Control Study
Source: Front Public Health. 2022 May 12;10:821905. doi: 10.3389/fpubh.2022.821905 (PMC9133471; doi:10.3389/fpubh.2022.821905)
Supplement: Supplementary Table 1 — The maternal age stratification (<30,≥30 years) subgroups were analyzed for the associations between ambient SO2 exposure(μg/m3) with controls and cases. [file Table_1.docx]

**Supplemental Table 1. The maternal age stratification (<30,≥30 years) subgroups were analyzed for the associations between ambient SO_2_ exposure(μg/m^3^) with controls and cases.**

| **Tertiles of SO_2_ level^a^** | **<30 years (4875)** | | | **Tertiles of SO_2_ level^a^** | **≥30 years (3367)** | | | **P for interaction** |
| --- | --- | --- | --- | --- | --- | --- | --- | --- |
|  | **No. of cases(171)** | **No. of controls(4704)** | **Adjusted OR^b^ (95% CI)** |  | **No. of cases(121)** | **No. of controls(3246)** | **Adjusted OR^b^ (95% CI)** |  |
| **The first month of pregnancy** | | | | | | | | 0.52 |
| <22 | 49 | 1612 | 1.00 (ref) | <21 | 38 | 991 | 1.00 (ref) |  |
| 22 to <40 | 53 | 1540 | 1.15 (0.73-1.81) | 21 to <41 | 36 | 1137 | 0.82 (0.48-1.39) |  |
| ≥40 | 69 | 1552 | 1.64 (0.90-2.97) | ≥41 | 47 | 1118 | 1.29 (0.64-2.61) |  |
| Per 1-SD increase |  |  | 1.16 (1.07-1.37) | Per 1-SD increase |  |  | 0.64 (0.41-0.97) |  |
| **The second month of pregnancy** | | | | | | | | 0.62 |
| <21 | 42 | 1533 | 1.00 (ref) | <21 | 31 | 1018 | 1.00 (ref) |  |
| 21 to <43 | 51 | 1628 | 1.44 (0.91-2.30) | 21 to <46 | 38 | 1113 | 1.35 (0.78-2.34) |  |
| ≥43 | 78 | 1543 | 2.75 (1.32-5.95) | ≥46 | 52 | 1115 | 2.48 (1.09-5.90) |  |
| Per 1-SD increase |  |  | 1.01 (0.74-1.36) | Per 1-SD increase |  |  | 1.19 (0.98-1.47) |  |
| **The third month of pregnancy** | | | | | | | | 0.42 |
| <23 | 49 | 1492 | 1.00 (ref) | <23 | 40 | 976 | 1.00 (ref) |  |
| 23 to <51 | 49 | 1656 | 1.10 (0.67-1.78) | 23 to <52 | 34 | 1150 | 0.69 (0.39-1.21) |  |
| ≥51 | 73 | 1556 | 1.86 (0.96-3.58) | ≥52 | 47 | 1120 | 0.99 (0.46-2.13) |  |
| Per 1-SD increase |  |  | 1.37 (1.09-1.86) | Per 1-SD increase |  |  | 1.03 (0.70-1.49) |  |
| **The first trimester** | | | | | | | | 0.21 |
| <24 | 45 | 1464 | 1.00 (ref) | <24 | 39 | 942 | 1.00 (ref) |  |
| 24 to <48 | 51 | 1717 | 1.46 (0.88-2.41) | 24 to <53 | 39 | 1204 | 0.69 (0.38-1.22) |  |
| ≥48 | 75 | 1523 | 3.14 (1.42-6.94) | ≥53 | 43 | 1100 | 0.65 (0.26-1.64) |  |
| Per 1-SD increase |  |  | 1.48 (1.25-1.93) | Per 1-SD increase |  |  | 1.14 (0.71-1.72) |  |

Abbreviations: CI,confidence interval; OR,odds ratios; SD,standard deviation; SO_2_,sulfur dioxide.

^a^ SO_2_ concentrations (μg/m³) are based on the monthly average concentrations, which are then averaged over different exposure windows and analyzed in tertiles (determined from controls).

^b^ Logistic regression analysis adjusting for season of conception, gravidity, parity, maternal education and maternal nitrogen dioxide and particulate matter with an aerodynamic diameter ≤ 10 μm exposures during the same exposure window.
